# A new model of multi-visceral and bone metastatic prostate cancer with perivascular niche targeting by a novel endothelial specific adenoviral vector

**DOI:** 10.18632/oncotarget.14699

**Published:** 2017-01-17

**Authors:** Zhi Hong Lu, Sergey Kaliberov, Rebecca E. Sohn, Lyudmila Kaliberova, Yingqiu Du, Julie L. Prior, Daniel J. Leib, Anne Chauchereau, Jennifer K. Sehn, David T. Curiel, Jeffrey M. Arbeit

**Affiliations:** ^1^ Urology Division and Department of Surgery, Washington University in St. Louis School of Medicine, St. Louis, MO 63110, USA; ^2^ Siteman Cancer Center, Washington University in St. Louis School of Medicine, St. Louis, MO 63110, USA; ^3^ Biologic Therapeutics Center, Washington University in St. Louis School of Medicine, St. Louis, MO 63110, USA; ^4^ Department of Radiation Oncology, Washington University in St. Louis School of Medicine, St. Louis, MO 63110, USA; ^5^ Radiology, Washington University in St. Louis School of Medicine, St. Louis, MO 63110, USA; ^6^ Department of Orthopedic Surgery, Washington University in St. Louis School of Medicine, St. Louis, MO 63110, USA; ^7^ Prostate Cancer Group, INSERM U981, Gustave Roussy, Villejuif, F-94805, France; ^8^ Department of Anatomic and Molecular Pathology, Washington University in St. Louis School of Medicine, St. Louis, MO 63110, USA

**Keywords:** prostate, metastasis, endothelial, niche, adenovirus

## Abstract

While modern therapies for metastatic prostate cancer (PCa) have improved survival they are associated with an increasingly prevalent entity, aggressive variant PCa (AVPCa), lacking androgen receptor (AR) expression, enriched for cancer stem cells (CSCs), and evidencing epithelial-mesenchymal plasticity with a varying extent of neuroendocrine transdifferentiation. Parallel work revealed that endothelial cells (ECs) create a perivascular CSC niche mediated by juxtacrine and membrane tethered signaling. There is increasing interest in pharmacological metastatic niche targeting, however, targeted access has been impossible. Here, we discovered that the Gleason 7 derived, androgen receptor negative, IGR-CaP1 cell line possessed some but not all of the molecular features of AVPCa. Intracardiac injection into NOD/SCID/IL2Rg ^-/−^ (NSG) mice produced a completely penetrant bone, liver, adrenal, and brain metastatic phenotype; noninvasively and histologically detectable at 2 weeks, and necessitating sacrifice 4-5 weeks post injection. Bone metastases were osteoblastic, and osteolytic. IGR-CaP1 cells expressed the neuroendocrine marker synaptophysin, near equivalent levels of vimentin and e-cadherin, all of the EMT transcription factors, and activation of NOTCH and WNT pathways. In parallel, we created a new triple-targeted adenoviral vector containing a fiber knob RGD peptide, a hexon mutation, and an EC specific ROBO4 promoter (Ad.RGD.H5/3.ROBO4). This vector was expressed in metastatic microvessels tightly juxtaposed to IGR-CaP1 cells in bone and visceral niches. Thus, the combination of IGR-CaP1 cells and NSG mice produces a completely penetrant metastatic PCa model emulating end-stage human disease. In addition, the metastatic niche access provided by our novel Ad vector could be therapeutically leveraged for future disease control or cure.

## INTRODUCTION

Despite enormous strides in therapeutic development, metastatic prostate cancer remains fatal. Treatment with abiraterone an inhibitor of cytochrome P450 17A1 (CYP17A1 17α-hydroxylase/17,20 lyase)-mediated androgen synthesis, or enzalutamide, which inhibits three androgen receptor (AR) functions; ligand binding, nuclear translocation, and DNA binding, have increased quality of life and life span. However, these more potent drugs and the newer taxanes, alone or in combination, also appear to foster an increasingly evident clinical and pathological entity, “aggressive variant prostate cancer (AVPCa)” [1–3]. AVPCa is accompanied by visceral (liver, adrenal, and brain) in addition to osseous metastases [4, 5]. AVPCa metastases possess a range of histological phenotypes, lack androgen receptor protein expression, and express a variable number of neuroendocrine markers [1, 6, 7]. The combination of visceral with bone metastases confers a poor prognosis and patients die soon after diagnosis [8–10].

While the proximate cause for AVPCa development is selection under AR signaling inhibition [3, 11], the target cell for this selection is likely the cancer stem/tumor initiating cell (CSC) [12]. This cell population is maintained by a collection of host cells termed the niche [13]. Malignant cell proliferative quiescence appears to require continuous suppression. Suppression is achieved by niche cell secretion of molecules and cell surface integrin display [14]. Metastatic niche signaling is predominantly short range. That is, molecules secreted by niche cells tend to either be membrane tethered, or stromal matrix bound. As niche signaling requires intimate interactions between malignant cells and host niche components it is not surprising that lineage tracing or immunofluorescence has revealed tumor-niche juxtaposition [15]. Niche cellular component predominance appears to vary, in part, in different host organs. In the bone, osteoblasts and mesenchymal cells are prominent [16]. However, one near universal niche component that appears to be to the principal arbiter of the proliferatively quiescent metastatic cell population is the vascular endothelial cell (EC). In bone marrow, ECs are one major component of the hematopoietic stem cell niche [17]. Perivascular niches also appear to play significant roles in metastatic cell quiescence in brain glioblastomas, breast metastases, and in hematological malignancies [15]. The relatively recent availability of a reliable endothelial targeted Cre recombinase transgenic mouse has enabled the genetic discovery of short range signaling ligands secreted by endothelial cells in response to damage, during development, and in malignant niches in both primary and metastatic cancer [18]. This short range signaling has been termed “angiocrine” [19]. Angiocrine functions have been shown as necessary for hematopoietic recovery following sublethal radiation or 5-fluorouracil chemotherapy in the bone marrow [19]. Endothelial secretion of a soluble form of the NOTCH ligand Jagged-1 was required for growth of colon liver metastases, and for lymphoma maintenance [20]. Given its potential importance in metastatic persistence and therapeutic recalcitrance the tumor-perivascular niche is an ideal candidate for therapeutic manipulation. Unfortunately, discrete access to this tissue compartment has been impossible other than in genetic mouse models.

Here, we have used a recently described PCa cell line, IGR-CaP1, that forms osteoblastic metastases following intratibial or systemic arterial injection [21]. We have discovered that IGR-CaP1 cells closely emulate aggressive PCa. Highly immunodeficient NOD/SCID/ILR2 γ^-/−^ (NSG) mice [22] evidence a 100% incidence of bone, liver, and adrenal experimental metastases, detectable by 2 weeks post intracardiac injection. IGR-CaP1 cells lack AR protein expression, while upregulating neuroendocrine markers. The cells appear to be in dynamic equilibrium between epithelial and mesenchymal fates, and express stem cell markers. They also appear to have extensive DNA damage. In parallel with model creation and molecular interrogation, we have constructed a novel endothelial-targeted adenoviral (Ad) vector that we genetically modified for enhanced peripheral uptake, diminution of liver hepatocyte sequestration, and enhanced tumor-associated endothelial expression. Systemic injection of this vector produces transgene reporter expression in the microvasculature immediately juxtaposed to metastatic cells in bone and visceral metastases. Thus, we now have both a mouse model and unprecedented access for testing the necessity for a growing list of signaling pathways thought to be crucial metastatic niche maintenance. These vectors could potentially be used as new standalone therapies or to pinpoint drug targets that could enable control or cure for otherwise lethal metastatic PCa.

## RESULTS

### Development of a completely penetrant, rapid onset, model of experimental bone and multi-visceral organ metastatic prostate cancer

**Figure 1:**
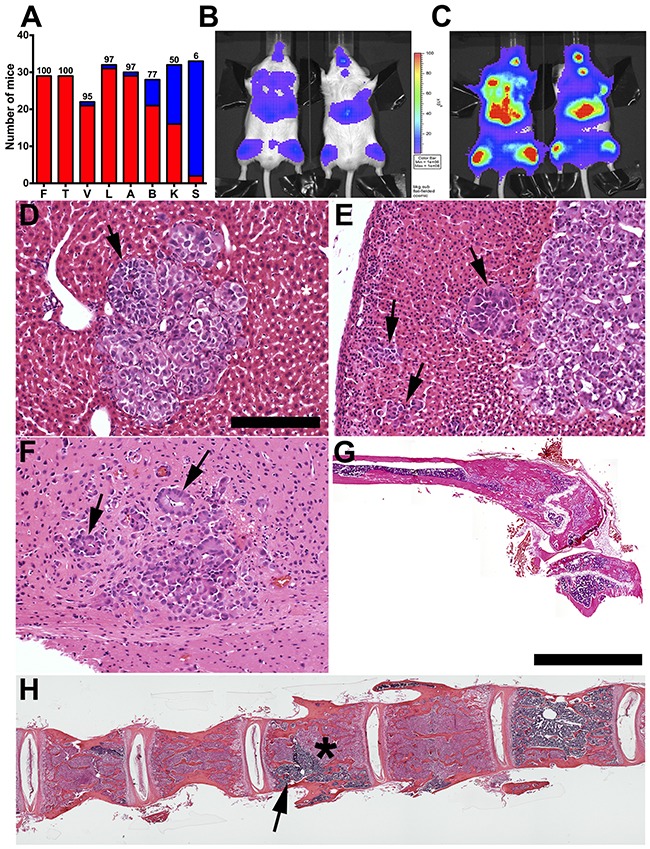
Incidence, kinetics, and histopathology of skeletal and solid organ metastases in NOD/SCID/IL2Rγ (NSG) immunodeficient mice **A**. Experimental metastases incidence percentage (above each column red is number of mice with detectable tumors, blue is mice with undetectable metastases) following left ventricular injection of 5×10^5^ IGR-CaP1 cells (n=29-32 mice). **B**. BLI signals were detectable in bone and visceral sites 2 weeks post intracardiac injection increasing in intensity at 4 weeks (vertical scale bar (photons/sec/cm^2^/steradian) is the same for B and **C**). **D**. Liver and **E**. adrenal metastasis (black arrows) with “pseudo-glandular” morphology (see also Supplementary Figure 1). **F**. Brain metastasis with well differentiated tumor cells arranged in a rosette-like morphology surrounding microvessels consistent perivascular pseudo rosettes (black arrows). **G**. Femoral metastases with extensive intramedullary new bone formation. **H**. Lumbar vertebral column metastases with variable tumor (asterisk) versus normal marrow (arrow) replacement. Compared to femur, **G**, there is less extensive new bone formation. Bars: D-F: 200 μm; G, H: composite images of multiple 4X fields, 4 mm.

In the original reports of the IGR-CaP1 cell line, nude (nu/nu) mice were the recipient immunodeficient hosts [21, 23]. However, the time for metastases to reach appreciable size, 7-9 weeks, and the bone metastatic incidence, 55%, motivated us to test mouse strains with greater degrees of immunodeficiency. Inspired by purported enhanced human cell receptivity of highly immunodeficient NSG mice, we tested IGR-CaP1 bone and visceral experimental metastatic frequencies following intracardiac injection [24]. To compare our work with the original studies of the IGR-CaP1 cell line, we injected the same number of tumor cells, 5×10^5^, into the left ventricle [21, 23]. To facilitate both noninvasive and fluorescence detection of microscopic metastatic foci, we created a new IGR-CaP1 cell line expressing both click beetle red luciferase and mCherry. BLI signals were detected at 2 weeks, increasing in intensity by 4 weeks post tumor cell injection (Figures 1B, C). Mice required sacrifice at 5 weeks post injection. Histopathology and fluorescence revealed that 97-100% of NSG mice evidenced liver, adrenal, and bone metastases (Figures 1A, D-H). Brain and kidney metastases were detected in 77 and 50% of mice respectively (Figure 1A, F, and kidney not shown). Bone metastases were detected in the most common regions affecting prostate cancer patients including the tibia, vertebral column, femur, humerus, maxilla and mandible (Figure 1B, C, G, H, and data not shown).

More detailed histopathological analysis revealed that IGR-CaP1 liver, kidney, and adrenal metastases were relatively well-circumscribed nodules of poorly differentiated carcinoma growing in sheets and nests with scant stroma and only focal gland-like spaces (Figure 1D, E, and Supplementary Figure 1). Occasional cells with more dense, eosinophilic cytoplasm, smudgy nucleoli and prominent cherry-red nucleoli also were observed, mostly distributed toward the periphery of tumor cell nests. Mitotic activity was brisk and included atypical forms. In the brain, groups of well differentiated metastatic cells surrounded microvessels, which is a structure similar to perivascular pseudo rosettes that are detectable in brain ependymomas (Figure 1F) [25]. At the time of sacrifice, 5 weeks post injection, metastatic liver and adrenal tumors were extensive replacing large areas of liver parenchyma, and nearly all of the adrenal gland. These “late stage” tumors evidenced multifocal single-cell as well as central “comedo” necrosis (Supplementary Figure 1B-C) [26]. In some areas, degeneration and necrosis in the tumor cell nests imparted a pseudopapillary architecture, though these areas lacked true papillae with fibrovascular cores (Supplementary Figure 1A, arrow). In addition, focal tumor gland formation was also detected (Supplementary Figure 1A, arrowhead). Extensive hepatic and adrenal metastases likely underlain the necessity for mouse sacrifice at this time point. Collectively, the IGR-CaP1/NSG experimental metastasis model was markedly accelerated compared the original reports wherein nu/nu mice were used as hosts [21, 23]. As genetic drift in our IGR-CaP1 cell stock could be one explanation for our metastatic frequency differential compared to the original report, we had STR chromosomal marker analysis done by an outside collaborator. Our stock evidenced the same markers as the prior study [23].

### IGR-CaP1 cells stimulate osteoblastogenesis, consistently forming mixed osteoblastic/osteolytic tumors

**Figure 2:**
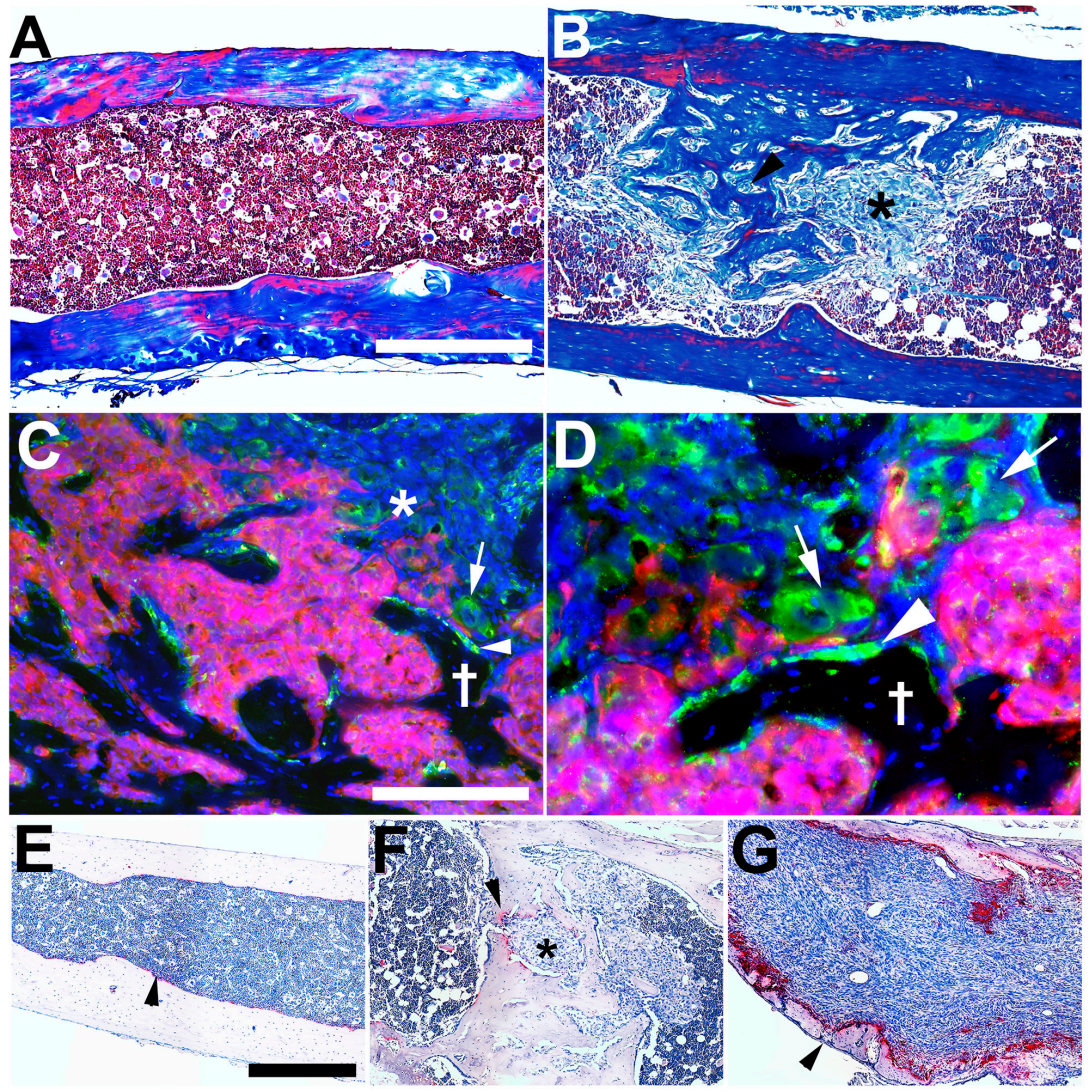
IGR-CaP1 cells create osteoblastic metastases at multiple skeletal sites **A**. Trichrome stain of tibial shaft in a nontumor bearing mouse. **B**. Trichrome stain of IGR-CaP1 metastases in tibia shaft revealing extensive intracavitary osteogenesis (asterisk and black arrowhead tumor cells, completely surrounded by new bone formation). **C**. Osteocalcin (osteoblast marker) immunofluorescence (**Green**) reveals osteoblast activation in juxtaposed to metastatic IGR-CaP1:CBRLUC-mCherry (**Red**) cells (arrowhead). There is also evidence for “osteomimicry” with osteocalcin expression evident in mCherry negative metastatic IGR-CaP1 cells (asterisk, and arrow) Latin cross: bone trabecula. **D**. Higher magnification view of **C**, to highlight osteoblast-tumor cell juxtaposition (arrowhead) and osteocalcin expression in mCherry negative and positive (arrows) IGR-CaP1 cells. **E**. TRAP stain (**Red-Pink**, **Blue**, hematoxylin nuclear counterstain) (osteoclast activity) of normal bone (arrowhead). **F**. Low level osteoclast activity (arrowhead) in an osteoblastic IGR-CaP1 metastasis (asterisk). **G**. Intense osteoclast activity in a renal cancer (786-O cells) osteolytic skeletal metastasis with microbreaks in the bone cortex (arrowhead). **Blue C, D**: DAPI. **Bars: A, B**: 400 μm; **C, D**: 200 μm, **E-G**: 100 μm.

To further explore the metastatic biology and the tumor-host niche interactions of experimental NSG host IGR-CaP1 metastatic tumors we used a combination of histopathology and molecular immunofluorescence analysis. Skeletal metastases were profoundly osteoblastic with marked new bone formation, particularly evident using Masson-trichrome staining (Figure 2A and 2B). To further investigate osteoblastogenesis, osteocalcin immunofluorescence was performed (Figure 2C and 2D). There were an increased number of osteocalcin positive cells with an osteoblastic morphology (Figure 2C and 2D, arrowhead and Latin cross). Metastatic IGR-CaP1 cells were detected in close juxtaposition to the osteocalcin positive cells. Scattered individual bone metastatic IGR-CaP1 cells also expressed osteocalcin (Figure 2C, arrow, magnified in 2D), suggesting the initial stages of osteomimicry, although IGR-CaP1 failed to mineralize in bone forming media in culture (data not shown). As both human data and prior work demonstrated that IGR-CaP1 bone metastases also stimulated osteoclastogenesis, we tested for tartrate-resistant acid phosphatase (TRAP) activity levels in our NSG-based model. In contrast to previous work [21], there was low-level increase in TRAP activity compared to normal bone, which was markedly decreased compared to the osteoclastogenesis and osteolytic activity of 786-O renal carcinoma cells (Figure 2E–2G).

**Figure 3:**
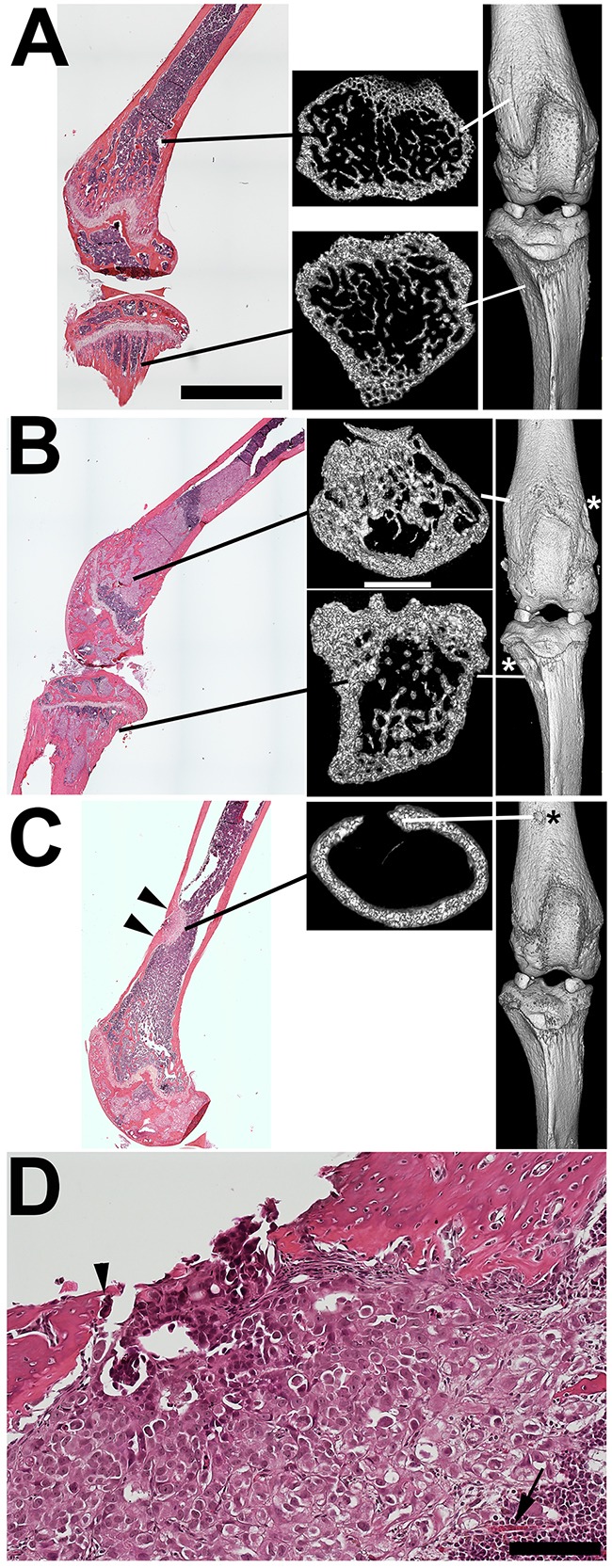
MicroCT reveals both bone remodeling consistent with metastatic tumor-mediated new bone formation and focal osteolysis **A**. Femur and proximal tibia from a non-tumor bearing NSG mouse with transaxial and 3D images. Note the fine trabecular structure of both metaphyseal regions. **B**. H and E staining reveals extensive tumor replacement of the femoral and tibial metaphysial regions in an IGR-CaP1 injected mouse. MicroCT reveals increased intramedullary bone volume in both regions. 3D rendering of these bones reveals cortical defects in both distal femur and proximal tibia (asterisk). **C**. Histological staining of another IGR-CaP1 injected mouse reveals a focal diaphysial cortical disruption with remodeling-mediated cortical thickening at each edge of the breach (arrowheads). Transaxial microCT of the same region showing the cortical disruption and thickening of one edge. 3D view also detected the cortical lesion (asterisk). **D**. Histology of the cortical disruption of C. Surprisingly, there is a paucity of osteoclasts (arrowhead) given the obvious osteolytic activity. An intramedullary artery is seen supplying the tumor (arrow). Bars: A-C (H+E sections): 4 mm. A-C (microCT): 1 mm; D: 100 um.

To globally investigate IGR-CaP1 cell alterations of bone morphology we used microCT analysis of tumor bearing (n=2 mice) versus non-tumor bearing (n=1) femur, tibia, and spine (Figure 3). 3D rendering of the entire femurs and tibias revealed multiple sites of cortical discontinuity particularly within or immediately adjacent to the metaphyseal regions of both bones at the knee (Figure 3B and 3C, white and black asterisks). Cross sectional analysis of the metaphyseal regions using transaxial 3D imaging revealed increases in metaphysial bone volume in both femur and tibia in the tumor bearing compared to the non-tumor bearing mice (Figure 3A and 3B). A circular discontinuity in the distal diaphysis of one of the femurs was also detected (Figure 3C). Histological step sectioning revealed that the cortical defect was filled, and likely caused by, a juxtacortical tumor mass (Figure 3D). These data indicative of osteolysis were surprising given the paucity of TRAP activity (Figure 2F). Transient focal increases in osteoclast activity could be one explanation for their infrequency in areas of cortical disruption (Figure 3D). In contrast to the femur and tibia metaphysial bone remodeling, there were no detectable abnormalities in the proximal femur at the hip, in the distal tibia at the ankle, and in the spine.

### IGR-CaP1 cells express a subset of molecules associated with aggressive PCa

**Figure 4:**
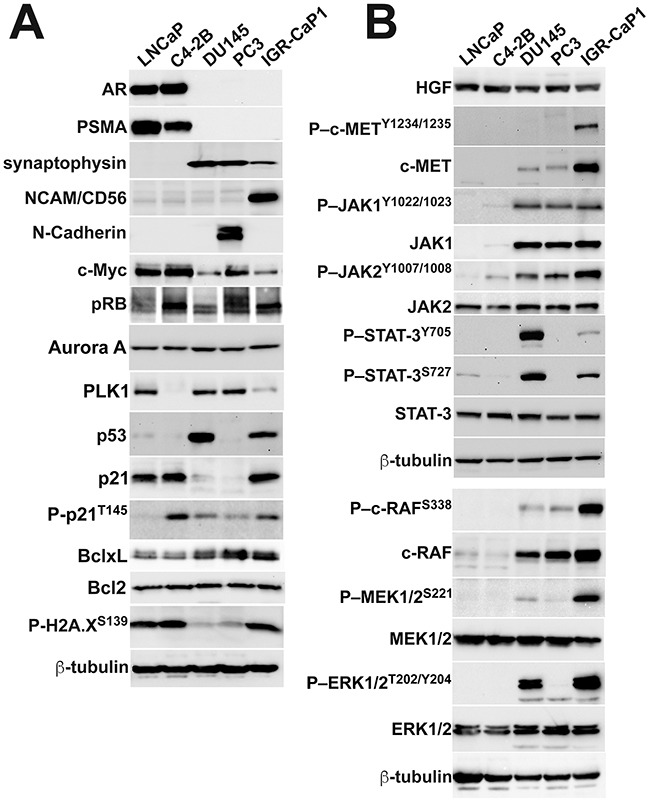
IGR-CaP1 cells express a subset of molecules coordinating the aggressive disease phenotype **A**. Robust AR and PSMA expression in LNCaP and C4-2B cells, with lack of expression of these proteins in IGR-CaP1, PC3 and DU145 cells. In contrast, the AR(–) cell lines including IGR-CaP1, express synaptophysin. IGR-CaP1 also evidence the greatest level of NCAM expression, but N-Cadherin is undetectable. IGR-CaP1 cells do not differentially express c-Myc, Aurora A, and polo-like kinase-1 (PLK1), and retain retinoblastoma protein expression. P53 protein is markedly expressed in both IGR-CaP1 and DU145 cells, while p21 expression is differentially elevated in IGR-CaP1, LNCaP and C4-2B cells. Phospho-p21 is easily detectable in all cell lines save for trace detectability in LNCaP cells. BclxL is differentially elevated in the AR(–) compared to the AR(+) cell lines, while Bcl2 is equivalently expression by all of the cell lines. Phospho-histone 2A (p-H2A.X^S139^) is differentially elevated in IGR-CaP1 and the AR(+) cell lines. **B**. HGF is equivalently expressed in all cell lines, but high-level phospho-c-MET^Y1234/1235^ and c-MET are markedly elevated in IGR-CaP1 compared to all other cell lines. AR(–) cells differentially express the downstream c-MET signaling components, phospho-JAK1 and JAK2 and phospho-STAT3^Y705/S327^ compared to AR(–) cells. Phospho-c-RAF (Ras interaction site), MEK, and ERK1/2 are expressed at the greatest levels in IGR-CaP1 cells compared to all other cell lines.

**Figure 5:**
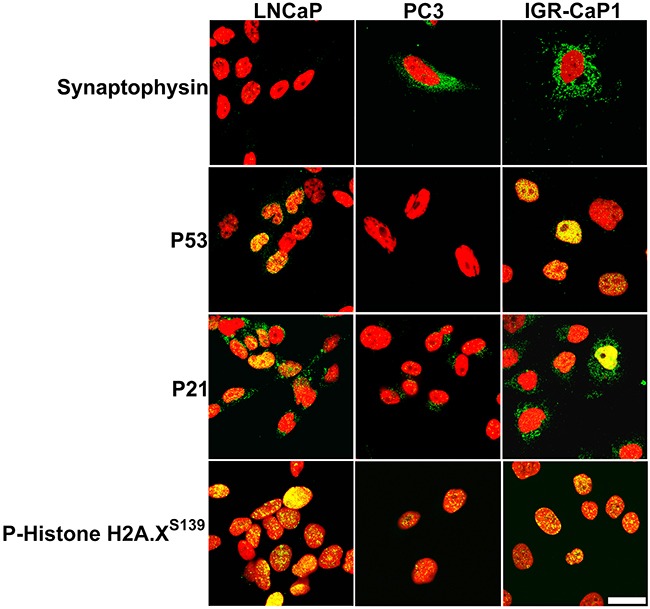
Intracellular localization of proteins detected by single slice, mid-nuclear plane, confocal microscopy of cultured IGR-CaP1 cells Synaptophysin protein is detected in vesicles throughout the cytoplasm in IGR-CaP1 and PC3 cells, but is not evident in LNCaP counterparts. p53 protein is nuclear localized and heterogeneously expressed IGR-CaP1 and LNCaP cells but absent in p53 counterparts. High level p21 expression is evident in IGR-CaP1 cells but is nuclear excluded in most cells. In contrast p21 is predominantly nuclear localized in LNCaP counterparts. Both IGR-CaP1 and LNCaP cells express high level, nuclear foci localized H2A.X^S139^. **Green**: each target protein, **Red**: TOPRO3. Bar: 20 μm.

The combination of dual bone and visceral organ metastases led us to question whether IGR-CaP1 cells modeled the emerging clinical entity, aggressive PCa at the molecular level [7]. Aggressive PCa fails to express androgen receptor (AR), displays varying degrees of neuroendocrine transdifferentiation, possesses distinctive oncogenic and tumor suppressor gene amplification or loss of function mutations, activates the DNA damage response, and upregulates signaling pathways stimulating proliferation [7]. First, we tested for IGR-CaP1 cell expression of AR and one of its target genes, prostate specific membrane antigen (PSMA), in a representative PCa cell line panel composed of known AR(+) cell lines (LNCaP, and its derivative, C4-2B), compared to AR(–) (DU145 and PC3) cell lines. Similar to the original report IGR-CaP1 cells were AR protein negative (Figure 4A) [23]. Testing for synaptophysin expression, a neuroendocrine marker, revealed data diametrically opposed to that of AR; that is the AR (+) cell lines, were synaptophysin negative, whereas AR(–) cell lines, including IGR-CaP1 cells, expressed synaptophysin (Figure 4A). Confocal microscopy revealed synaptophysin localization in multiple vesicles dispersed throughout the cytoplasm in both IGR-CaP1, and PC3 cells, which were the positive controls in this experiment (Figure 5). Further immunoblotting for neuroendocrine markers revealed that CD56/NCAM was solely detectable in IGR-CaP1 compared to all other interrogated PCa cell lines including PC3 and DU145 (Figure 4A). In contrast, IGR-CaP1 cells did not detectably express other markers of neuroendocrine transdifferentiation, such as N-Myc (data not shown), or N-Cadherin, the latter was expressed in PC3 cells (Figure 4A). In addition to neuroendocrine transdifferentiation, other molecular features such as apoptosis resistance, loss of retinoblastoma protein (pRB), and gene amplification of Aurora, polo-like kinases (PLK1), and c-Myc, also coordinate the aggressive PCa phenotype [7, 27]. IGR-CaP1 cells possessed one of these attributes, overexpression of the anti-apoptotic BclxL protein (Figure 4A). However, IGR-CaP1, and the remainder of our AR(+) and AR(–) cell lines did not overexpress c-Myc, Aurora A, or PLK1, and they retained retinoblastoma protein expression (Figure 4A). IGR-CaP1 immunoblotting and ICC revealed constitutive high level, nuclear localized p53 expression consistent with the original characterization of these cells (Figures 3A and 4) [23]. ICC also suggested heterogeneity with cells containing high level nuclear p53 expression and other cells wherein p53 protein was solely detected in nuclear foci (Figure 5). LNCaP cells displayed even greater p53 protein heterogeneity with many cells lacking detectable protein expression. As the p53 mutation reported for IGR-CaP1 cells, Y126C [23], has been shown to lack transcriptional activity [28], it was surprising that the p53 target, p21, was coordinately upregulated in IGR-CaP1 cells (Figure 4A). However, further investigation using ICC revealed that p21 was localized to the cytoplasm in the majority of IGR-CaP1 cells (only 19/394 cells positive for nuclear p21 expression) (Figure 5, representative high magnification confocal image). One explanation for these data is enhanced phosphorylation of p21 at threonine 145, which sterically hinders p21 nuclear translocation [29]. Indeed, T145 phosphorylation was present in IGR-CaP1 cell protein extracts (Figure 4A). In contrast, p21 was predominantly localized to the nucleus of LNCaP cells, which also expressed high levels of p53 and p21, but low levels of phosphorylated p21^T145^ (Figures 4A and 5). Proliferative quiescence could be one explanation for p21 cytoplasmic retention. However, the fact that most IGR-CaP1 cells expressed nuclear Ki67 (Supplementary Figure 2) obviated that explanation. The loss of cell cycle control suggested by the Ki67 and p21 expression levels and localization suggested that IGR-CaP1 cells were under oncogenic stress [30]. Elevated levels of gamma-H2A.X^pS139^ detected by immunoblotting, and its nuclear foci localization by ICC, supported that hypothesis (Figures 4A and 5).

Given the hyperproliferative and oncogenic stress phenotype of the IGR-CaP1 cells, we interrogated expression of proteins belonging to signaling pathways stimulating or regulating proliferation, and additionally associated with AVPCa (Figure 4B). One of these signaling modules, HGF-c-MET, is frequently overexpressed in AVPCa and AR inhibitor treated cancers [31]. HGF was expressed at similar levels in all our interrogated PCa cell lines. In contrast, detec-MET protein expression was restricted to AR(–) cells, with a marked differential elevation in IGR-CaP1 cells compared to other AR(–) counterparts. In addition, phosphorylated c-MET^Y1234/1235^, the initial site activated following ligand-induced oligomerization and an activator of receptor kinase activity, was solely detectable in the IGR-CaP1 cells, suggesting that the marked receptor overexpression sensitized these cells to (autocrine) growth factor stimulation. Additional support for elevated c-MET-mediated signaling in IGR-CaP1 cells, was provided by the differentially elevated JAK–STAT3, and markedly elevated c-RAF/MAPK kinase pathway phosphorylation, both downstream of c-MET receptor activation (Figure 3B). While other growth factors and RTKs could stimulate the MAPK kinase pathway, we did not detect differential EGFR or PDGFRβ phosphorylation (data not shown). Collectively, these data suggest that IGR-CaP1 cells possessed some but not all of the molecular attributes of AVPCa.

### IGR-CaP1 cells possess an EMT transition cell phenotype

**Figure 6:**
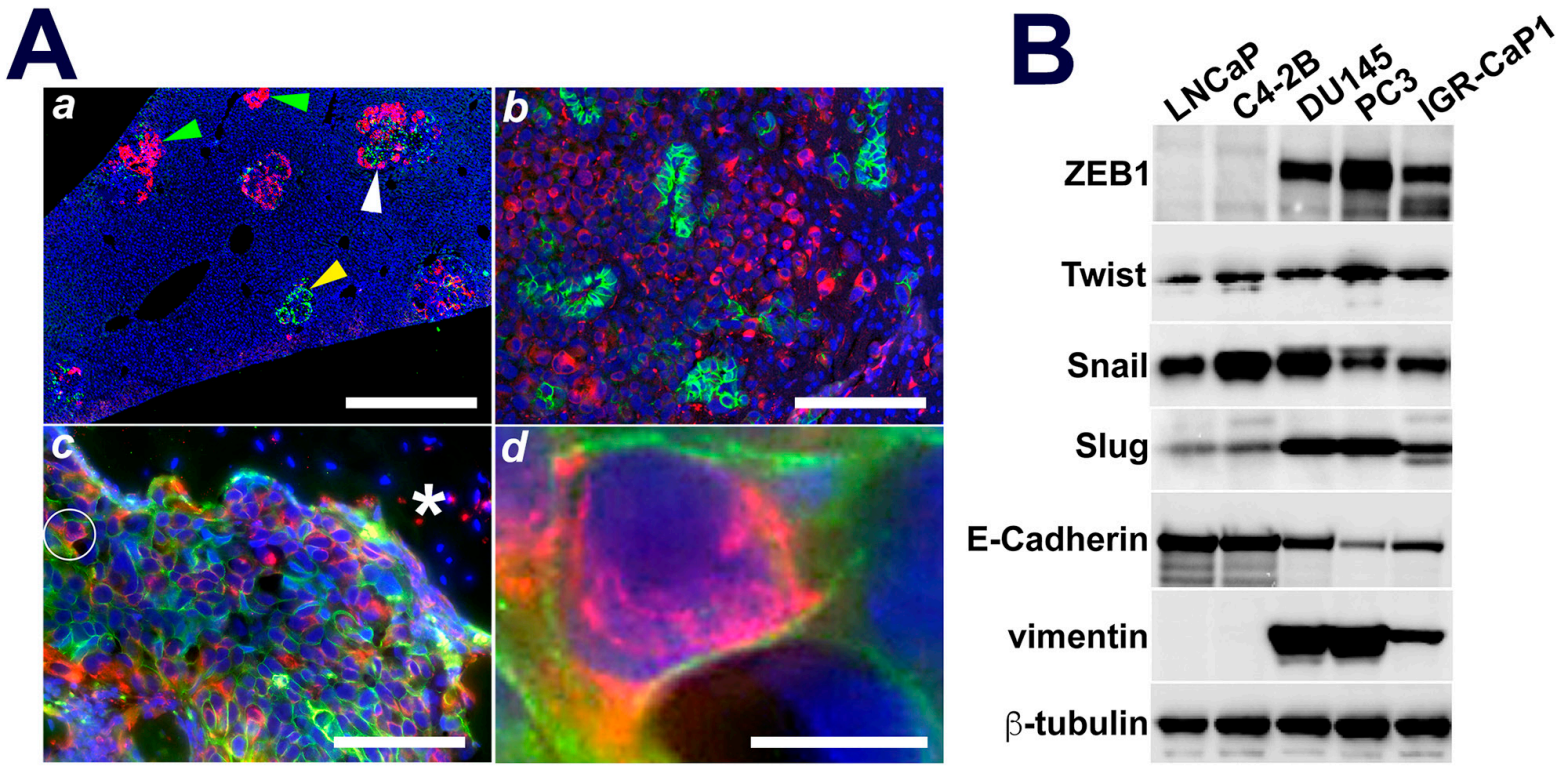
IGR-CaP1 metastatic subpopulations express organ dependent mixtures of epithelial or mesenchymal cell clusters or EMT in-transit cells **A**. Liver, brain, and adrenal metastases, **A*a*, A*b***, Distinct clusters of E-cadherin (yellow arrowhead) or vimentin (green arrowheads) positive cells either as solo tumors or adjacent to sheets of vimentin expressing cells in liver and brain metastatic tumors. **A*c***. Bone metastases contain an admixed population of E-cadherin and vimentin positive cells, most of which are EMT transition cells (white circle). **A*d***. EMT transition cell cropped and enlarged from **A*c*** wherein the plasma membrane localized E-Cadherin and cytoplasm-localized vimentin is evident. **B**. AR(–) cells evidence differentially elevated EMT transcriptional regulators, ZEB1 and Slug, with essentially equivalent Twist across all cell lines. Vimentin was solely detectable in AR(–) cells, while E-cadherin was downregulated but still detectable in AR(–) compared to robust expression in AR(+) cells. IGR-CaP1 cells expressed near equivalent E-cadherin and vimentin proteins, while PC3 and DU145 cells massively overexpressed vimentin compared to E-cadherin; consistent with the EMT transition phenotype of IGR-CaP1 cells. **Green**: E-Cadherin; **Red**: vimentin; **Blue**: DAPI. **A*c***: Asterisk is bone trabecula. **Bars**: **A*a***: 1 mm, **A*b***: 200 μm, **A*c***: 100 μm; **A*d***: 5 μm.

As IGR-CaP1 cells were reported to be enriched for cancer stem cell (CSC) activity and marker expression [23], and since CSCs presumably underlie the development of pan-therapeutic resistance in AVPCa [32], we explored this further in our NSG mouse model. Epithelial-mesenchymal transition (EMT) has been shown to be a CSC function [33]. Therefore, we tested for evidence of EMT using E-cadherin and vimentin immunofluorescence (Figure 6). In liver and brain metastases, distinct clusters of IGR-CaP1 cells were positive for either E-cadherin or vimentin, while cells expressing both proteins were sporadically detectable (Figure 6Aa and 6Ab). In contrast, IGR-CaP1 bone metastases were mainly comprised of cell possessing both plasma membrane-localized E-cadherin and cytoplasmic vimentin (Figures 6Ac and 5Ad), suggesting that the bone microenvironment might promote an EMT transition phenotype [34]. To test for molecular evidence of EMT, we immunoblotted our PCa cell line panel for expression of E-Cadherin, vimentin and a collection of EMT coordinating transcription factors. All of our AR(–) and AR(+) PCa cell lines expressed E-cadherin protein, with greater expression levels in the latter compared to the former (Figure 6B). Vimentin was solely detectable in AR(–) PCa cell lines (Figure 6B). Both DU145 and PC3 cells expressed massive levels of vimentin compared to E-cadherin. In contrast, the expression ratio of these two proteins in IGR-CaP1 cells was approximately equivalent, with a slight predominance of vimentin (Figure 6B). Expression of the master EMT transcriptional regulator, ZEB1, was abundant and restricted to AR(–) cell lines. Slug (SNAIL2) expression was also differentially elevated in AR(–) cell lines compared to low-level expression in AR(+) lines, while Twist was equivalently expressed in each cell line independent of AR status (Figure 6B). While this work validates the IGR-CaP1 cell EMT transition phenotype, additional experiments will need to be done to delineate the mechanisms maintaining this intermediary state in these cells.

### Activation of CSC-related developmental pathways in IGR-CaP1 cells

**Figure 7:**
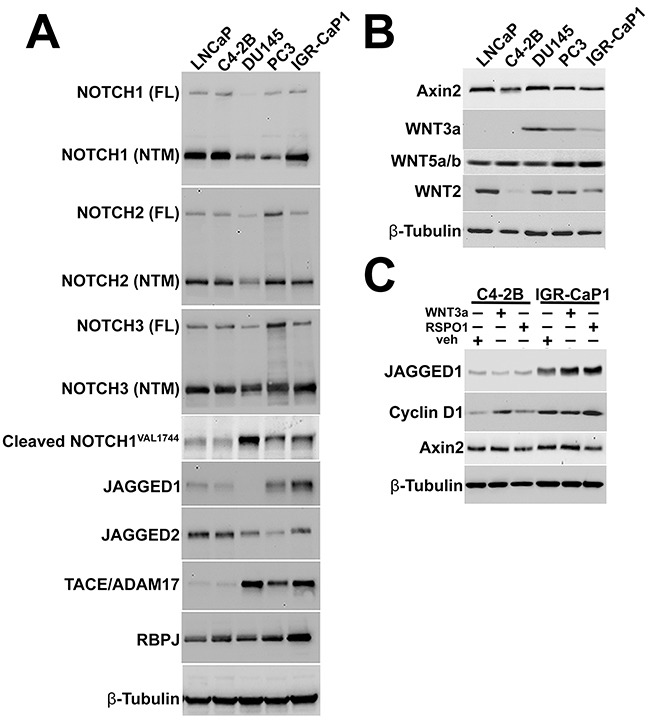
NOTCH and WNT pathway activation in IGR-CaP1 cells **A**. IGR-CaP1 cells express NOTCH receptors 1-3. The IGR-CaP1, PC3, and DU145 cells express higher cleaved, activated NOTCH1 intracellular domain (NICD) protein than LNCaP and C4-2B cells. IGR-CaP1 cells express differentially elevated Jagged-1, and the NICD transcriptional effector, RBPJ expression compared with all other cell lines, whereas the TACE/ADAM17 protease is overexpressed in each AR(–) cell line. **B**. Detectable WNT 3a expression is restricted to the AR(–) cell lines, while the non-canonical ligand WNT5a/b is differentially elevated in IGR-CaP1 cells. **C**. WNT target gene response to recombinant human (rh) WNT3a or R-spondin1 (RSPO1) stimulation in IGR-CaP1, compared to C4-2B cells. Jagged1 expression is solely increased by both rhWNT3a and rhRSPO1 in IGR-CaP1 cells.

As EMT can be a CSC precursor, IGR-CaP1 metastases were interrogated for molecules and signaling pathways known to regulate CSC niche maintenance and adherence. As both NOTCH and WNT have been shown to maintain the CSC phenotype in several types of cancer [35], including prostate [36, 37], we examined these pathways in cultured IGR-CaP1 cells. We discovered that each of the AR(+) or negative cell lines evidenced NOTCH1-3 receptor expression, with differentially elevated levels of the gamma secretase cleaved functional NOTCH intracellular domain transcription factor expression in the AR(–) cell lines (Figure 7A). In contrast, marked Jagged-1 ligand expression was uniquely detected in IGR-CaP1 cells compared to the other cell lines in our panel. At least three WNT ligands, WNT3a, WNT5a/b, and WNT2, were expressed in IGR-CaP1 cells (Figure 7B). Of interest, the non-canonical ligand, WNT5a/b, was expressed at the highest level in IGR-CaP1 cells. To further explore WNT pathway activity, we tested the responsiveness of IGR-CaP1 cells and the cell line most closely mimicking their bone metastatic phenotype, the LNCaP derived C4-2B cells, to either WNT3a or R-spondin-1 (RSPO1) stimulation; the latter molecule reported to be enriched in bone marrow stroma (Figure 7C) [25]. Both WNT3a and RSPO1 elevated Jagged-1 expression in IGR-CaP1 cells. In contrast, Jagged1 expression was not upregulated by either WNT3a or RSPO1 in C4-2B cells. As Jagged1 is a validated β-catenin target, these data are consistent with WNT pathway hyperresponsiveness in the IGR-CaP1 cells [38]. Finally, to further investigate expression of molecules associated with CSCs, we tested for CD44, CXCR4, and SDF1 expression in tissue sections from experimental IGR-CaP1 metastases. Both CD44 and CXCR4 were upregulated and plasma membrane localized in IGR-CaP1 metastases in skeletal and visceral organs (Supplementary Figure 3). SDF1 expression was markedly induced specifically in the tumor cells within each of the organs in our analysis. In bone marrow, the cell type specific expression pattern was more complex, SDF1 was differentially overexpressed in tumor cells, but also detectable in sinusoidal arteries and reticular stromal cells (Supplementary Figure 3).

### RGD.H5/3.ROBO4 Ad vector is endothelial cell specific with a tumor endothelial versus host organ expression bias

As AVPCa is the therapeutic terminus of widespread metastatic disease, new approaches to targeting resistance fostering niches are desperately needed for this increasingly frequent patient cohort [3]. The crucial contribution of endothelial cells (ECs) to metastatic niche maintenance, reported in other types of malignancies [20], led us to expand our prior work on endothelial transductional and transcriptional targeting of Ad vectors [39], with the goal of first testing for differential localization of vector expression in metastatic PCa as opposed to host vasculature targeting.

**Figure 8:**
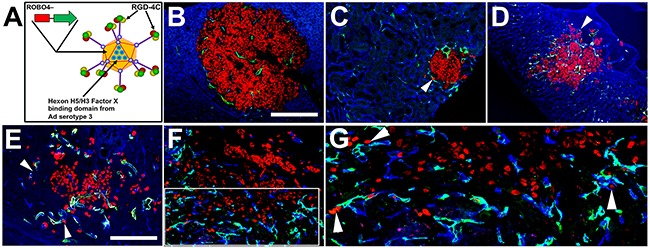
“Triple targeted” adenoviral vector containing three mutations designed to affect three aspects of adenoviral gene delivery in metastatic tumors **A**. A cyclized RGD peptide was inserted into the HI loop of the fiber knob. Liver “detargeting” was achieved by swapping the wild type coagulation Factor X capsid hexon binding site for the equivalent, though nonbinding, serotype 3 hexon region (H5/H3). The ROBO4 enhancer/promoter (3 kb) produces endothelial cell (EC) specific expression with a bias for tumor compared to normal organs. **B**. Liver metastasis with minimal vector expression in surrounding liver parenchyma. **C**. Kidney metastasis. **D**. Adrenal metastasis. **E**. Brain metastasis. **F**. Tibial shaft metastasis. **G**. Cropped, enlarged image from E (white rectangle). Single and clusters of IGR-CaP1:H2B/RFP cells and juxtaposed to the EC abluminal surface of Ad.RGD.H5/3.ROBO4 expressing vessels, particularly in kidney, adrenal, brain, and bone metastases (white arrowheads) (organs and bones were harvested from mice 3-4 days post intravenous injection). **Red**: IGR-CaP1:H2B/RFP; **Green**: RGD.H5/3.ROBO4-EGFP; **Blue**: CD31/endomucin cocktail, blue background in liver are hepatocytes. Bars: **B-D, F**: 400 μm, **E, G**: 200 μm.

Previously, we had created and tested an endothelial cell (EC)-targeted Ad vector containing 3 kb of the ROBO4 enhancer/promoter [39]. While transcriptionally targeted to vascular endothelium with a tumor microvessel bias, this vector required warfarin depletion of the coagulation Factor X for significant tumor vascular delivery [39]. As warfarin could be contraindicated in metastatic PCa in general, and particularly in aggressive disease with liver metastasis, we created a new vector that would be “detargeted” from hepatic sequestration independent of pharmacological coagulation factor depletion (Figure 8A). As in previous work, we swapped the wild type hexon Factor X binding site amino acid sequences for those from Ad serotype 3 [40]. In addition, prior work has repeatedly demonstrated the infection (transductional) tropism of a cyclized RGD-4C peptide fiber/knob HI loop addition for either tumor cells (direct injection) or endothelial cells (systemic injection) [41]. As such, we created our final Ad vector, RGD.H5/3.ROBO4 that uniquely incorporates three crucial facets of: enhanced tumor EC adhesion (fiber/knob RGD display), augmented extrahepatic gene payload delivery (capsid hexon serotype swap), and tumor microenvironment-induced transcriptional upregulation (EC-specific ROBO4 enhancer/promoter) (Figure 8A).

As the major clinical challenge is systemic control or cure of multi-organ metastatic disease, we tested the RGD.H5/3.ROBO4-EGFP reporter vector administered intravenously to NSG mice bearing IGR-CaP1 experimental bone and visceral metastases 4 weeks post intracardiac administration. For these experiments, we created an IGR-CaP1 cell line constitutively expressing a histone 2B-red fluorescent protein (H2B-RFP) reporter. Intense RFP fluorescence, mediated by chromatin condensation, facilitated single cell metastatic detection (Figures 8B-8G). Expression of our triple-targeted Ad vector was evident in most of the microvessels adjacent to and within tumor metastases in liver, kidney, adrenal, brain, and bone (Figure 8B–8G). Single metastatic IGR-CaP1 cells were discovered intimately associated with the abluminal surface of RGD.H5/3.ROBO4-EGFP vector expressing ECs, particularly in the kidney, adrenal, brain and in the bone marrow (Figures 8C-8G). We also performed a comparative analysis of the expression extent of the RGD.ROBO4 Ad vector in a host organ panel in nontumor bearing mice (Supplementary Figure 4). Expression was detected in liver, adrenal, lung, and throughout normal bone marrow. Notably, skin, heart, kidney cortex, brain, intestinal vasculature did not evidence detectable Ad vector expression. Our genetic strategy and data as described above motivated us to designate this new vector as being “triple targeted” because its three genetic alterations enhance tumor endothelial uptake, evade liver sequestration, and augment tumor endothelial expression.

## DISCUSSION

There are three crucial aspects of our study. Using the combination of the highly immunodeficient NSG mouse with the IGR-CaP1 cell line we have created a new and highly penetrant preclinical model of metastatic prostate cancer. Both the concomitant visceral and osseous metastases and our protein expression profiling of this model strongly suggested that it emulated some, but not all the biological and molecular features of AVPCa, which is increasingly the final stage of disease progression in the modern era of potent androgen blockade and chemotherapy [1, 2]. Pan-therapeutic resistance of AV PCa and its increasing frequency demand new treatment strategies [42]. As such, our creation of a “triple-targeted” Ad vector enabling access to metastatic niches via EC tropism, offers new possibilities to therapeutically manipulate the perivascular microenvironment either to eliminate malignant cells when used alone, or to break pan-resistance and reestablish responsiveness in combination with conventional treatments.

A particularly outstanding feature of the model was exquisite osteotropism of the IGR-CaP1 cells in NSG hosts following intracardiac injection. Bone metastatic modeling can also be produced using direct intratibial injection [43]. While this approach preserves the opposite limb enabling a contralateral control in the same mouse, it induces an injection-activated wound reaction, fails to model circulating tumor cell implantation, and lacks extraosseous disease. Moreover, the extensive osteoblastic phenotype of the IGR-CaP1 cells, also reported in the prior work with these cells was striking [21]. However, in contrast to the previous studies, our approach of whole tissue histological imaging and fluorescence based marked delineation, provided a definitive picture of the extent of this process, and the alterations these cells induced in the bone marrow. Osteoblastic metastatic disease was thought to be rare in mouse models and certainly spontaneous bone metastases are rare in genetically engineered mice (GEM) [44]. However, starting with the derivation of the LNCaP subline, C4-2B [45], an increasing number of patient cell lines have been reported to evoke osteoblastic metastases in mice [46, 47]. In fact, the extensive intracavitary new bone formation we detected was similar to the patient derived MDA-PCa2b cell line and the more recent serially transplantable MDA118b xenograft line, both of which were created by the same group [46, 48]. While the MDA-PCa2b cells retain AR expression (albeit mutant AR), the MDA118b xenografts lack AR expression similar to IGR-CaP1 cells. One surprising feature of the IGR-CaP1/NSG model was the modest microCT evidence for osteosclerosis despite extensive histological new bone formation. Most likely, the extensive metastatic tumor replacement of liver and adrenal glands is responsible for the rapid lethal progression of the model, which prevents sufficient new bone mineralization in metastatic tumors. Future work will focus on derivation of new IGR-CaP1 cell lines isolated from bone metastases, as described in other PCa cell line models, with the goal of extending survival (see below) to achieve greater bone remodeling versus solid organ metastatic tumor growth [45].

While the accelerated metastatic growth of the IGR-CaP1/NSG model offers the advantages of rapid phenotypic screening for genetic manipulations, it fails to recreate the usual pace of the slow progression of human metastatic prostate cancer. IGR-CaP1 cells were derived from an intermediate stage, Gleason 7 primary prostate cancer. During the serial cell passaging necessary for cell line establishment, they obviously were selected for loss of AR expression. AR expression loss could be due to outgrowth of an AR negative cell present in the primary cancer [49, 50], or loss of AR expression during cell culture in androgen-depleted medium [51]. Moreover, our study has shown that they evidence both a transition EMT molecular and immunofluorescent profile elements of epithelial plasticity. Of interest, epithelial plasticity has been shown to facilitate bone metastases in general [34, 52], and in PCa in particular [53]. Collectively, the IGR-CaP1/NSG mouse model appears to closely emulate the increasingly evident, treatment failure related, clinical entity, AVPCa) [7, 27, 54, 55]. Similar to IGR-CaP1/NSG mice, AVPCa patients suffer from both osseous and multi-visceral metastases [5]. The addition of visceral organ spread has been shown to be rapidly lethal in patients [8, 9]. Thus, the rapid time course of IGR-CaP1/NSG mouse experimental metastases is entirely consistent with the clinical time course of AVPCa.

The other compelling facet of IGR-CaP1 cells is that they shared some, but not all, of the molecular attributes of AVPCa. Detection of an IGR-CaP1 neuroendocrine marker subset had not been reported in the prior work with these cells [23]. The elevated levels of nuclear localized p53, c-MET and activation of its downstream signaling outputs were also consistent with aggressive PCa, and loss of AR function [7]. The combination of enhanced cell cycle activity, evidenced by near universal Ki67 expression with the predominant frequency of p21 nuclear exclusion suggested that these cells possessed considerable cell cycle dysregulation. As loss of cell cycle control produces oncogenic stress [56], it was not surprising that IGR-CaP1 cells evidenced gamma-H2A.X upregulation consistent with extensive DNA double strand breaks [57]. In addition, the apparent recruitment of the NOTCH and WNT developmental pathways was also consistent with expression profiling of end-stage metastatic disease [58, 59]. However, other molecular attributes of AVPCa, in particular overexpression of c-Myc, Aurora A, N-Myc, and PLK1 and loss of retinoblastoma expression were not evident in IGR-CaP1 cells [7, 27].

Another aggressive disease hallmark is EMT [58]. IGR-CaP1 metastases appeared to be on the cusp of this transition with some tumors displaying an epithelial while other deposits a mesenchymal phenotype as evidenced by E-cadherin versus vimentin expression. Individual in transit cells expressing both molecules, albeit in distinct plasma membrane versus intracytoplasmic compartments were also prominent in bone metastases. This “hybrid” epithelial/mesenchymal phenotype has been described in both breast and prostate models [34]. Overexpression of the master EMT transcription factor, ZEB1, along with Slug, Snail, and Twist were additional support for an EMT program in IGR-CaP1 cells [61]. Intriguingly, and consistent with the IF images, the near equivalent E-cadherin and vimentin levels reinforced their transition status. Importantly, hybrid EMT cells appear to impart cancer stem cell plasticity thus facilitating a continuous generation of therapy resistant metastatic cell populations [62]. Collectively, the visceral and bone target organ proclivity, protein expression, signaling pathway, and hybrid EMT data, are consistent with cells and tumors that have crossed the aggressive disease threshold, but have not fully attained all of its features [7]. The enhanced likelihood of concomitant host toxicities mediated by systemic targeting of multiple cell cycle regulatory or stem cell maintenance pathways, provides compelling rationale for our efforts at EC-focused metastatic niche targeting.

The induction of multiple cell signaling and fate determination pathways evident in aggressive PCa highlights its associated therapeutic challenges. While small molecule and chemotherapeutic cocktails have successfully inhibited growth of subcutaneous xenografts, the clinical application of this approach could be fraught with toxicities particularly in susceptible organs with rapid cell turnover such as intestine, skin, and bone marrow. While the target repertoire of small molecule inhibitors is increasingly being narrowed, targeted delivery of therapeutics to specific cellular components of the metastatic microenvironment could obviate host toxicity, but enhance growth inhibitory efficacy. One approach is manipulation of vasculature to usurp EC angiocrine function [39]. This could be achieved via viral vector gene therapy. Disease-specific vascular endothelial cell targeting following systemic vector administration has been a long sought after goal in gene therapy [63]. The endothelium is the first contact cell layer during intravenous injection, offering the opportunity for “first pass” cell infection. However, endothelial cells express low to undetectable levels of the principal adhesive receptor for serotype 5 Ad vectors, Coxsackie adenovirus receptor (CAR) that is the gene therapy “workhorse”. Specificity for diseased versus normal host endothelium has been a challenge. One solution has been insertion of candidate or phage display selected peptides onto the fiber knob [64]. These insertions have been enabled by the presence of the HI loop in the knob protein structure [64]. The HI loop projects perpendicular to the fiber knob and is of sufficient length to accommodate peptide insertion. The endothelial selectins have been one peptide class inserted into the fiber knob, motivated by upregulation of this molecule in vessels in response to inflammatory environments both in benign diseases and in tumors [65]. The other commonly used peptide is the αvβ3 or αvβ5 integrin binding fragment, asparagine (R), glycine (G), aspartate (D). Cyclization of this peptide has been shown to markedly increase binding and peptide stability and that is the form inserted into the Ad vector HI loop [66]. Many tumor histotypes also upregulate these integrins, and RGD-displaying Ad vectors have been used for direct intratumoral injection, with recent impressive anti-tumor responses [67]. The other strategy is transcriptional targeting using enhancer/promoter elements activated in tumor endothelium [68]. There has been a plethora of DNA regulatory elements used in these vectors. Similar to transductional peptides, the focus has been on enhancer/promoters activated in tumor endothelial cells. The two most intensively studied have been a human VEGR2 promoter fragment or a composite, modular, pre-proendothelin enhancer promoter (PPE-1-3x) [68, 69]. Both elements, but particularly the PPE-1-3x promoter, are induced by hypoxia commonly present within most tumor microenvironments. This latter vector, now named VB-111, has been tested in Phase I trials [69]. In all cases, the goal of this work has been microvessel ablation. One challenge to this exhaustively investigated field has been a paucity of data on the multiplicity of vector expressing tumor endothelial cells. Moreover, stringent efforts to detect the distribution of number of nontumor bearing host organs expressing vascular targeted vectors have been limited.

Here, we created a new adenovirus incorporating three genetic modifications designed to address three challenges of systemic vector administration; first pass target organ infection, hepatocyte sequestration, and cell type specific gene expression. The cyclized RGD peptide was used for enhanced tumor vessel infection/transduction. A serotype 3 domain was swapped into hexon replacing the native serotype 5 correspondent to obviate coagulation Factor X binding mediating hepatocyte sequestration. Biased tumor vascular endothelial expression was achieved with the use of the human ROBO4 promoter [39]. This promoter is both hypoxia responsive, and contains an ETS binding element that likely facilitates transgene expression in tumor-activated endothelium [70]. This RGD.H5/3.ROBO4 vector produced widespread intratumoral vascular expression. While host vessel expression was still evident in a delimited organ set, this vector was universally expressed in metastatic tumor niches, strikingly so in the bone marrow. Residual host vessel expression could be this vector's Achilles heel. However, host toxicity likely rests on the targets of the vector payloads. Our present focus will be on expression of secreted protein traps for ligands maintaining the metastatic niche. There is evidence for differential sensitivities of tumor versus host stem cells for small molecular niche mobilizing drugs [71]. Whether this will also be true for our vectors remains to be investigated. That said, we are also constructing next generation vectors, based on the RGD.H5/3 platform, containing enhancer/promoter elements that potentially possess greater tumor vascular specificity. As systemic vector administration has been repeatedly demonstrated to be safe in humans, the field of vascular targeting is being rejuvenated. The strategy of vector-mediated perivascular niche eviction now offers the exciting promise, still unproven, for therapy of the most recalcitrant and lethal form of PCa malignancy.

## MATERIALS AND METHODS

### Adenoviral vector construction

Replication incompetent RGD.H5/3.ROBO4-EGFP adenovirus was created using a two-plasmid rescue method, as described previously [39]. Details about vector construct are provided in Supplementary Methods.

### Cell culture

Human prostate IGR-CaP1 cells were a generous gift from Anne Chauchereau, Institut Gustave Roussy (Villejuif, F-94805, France). STR analysis by an independent laboratory confirmed their maintenance of the originally reported profile (data not shown), thus serving as validation of this cell line. LNCaP, PC3, DU145 cell lines were obtained directly from ATCC. The LNCaP derivative C4-2B cells were obtained from Christopher Maher at WUSTL. Details of cell line propagation and RSPO or WNT3a stimulation experiments are available in Supplementary Data.

### Mouse model

Experimental procedures involving mice were carried out under a protocol approved by the Washington University Animal Studies Committee. Immunodeficient NOD.Cg-Prkdc^scid^ Il2rg^tm1Wjl^/SzJ (NSG) mice (The Jackson Laboratory, Stock No: 005557) were inbred in Washington University School of Medicine aseptic barrier mouse facility. To establish experimental tumor metastasis, NSG mice were anesthetized and injected with 5×10^5^ parental, H2B-RFP-labeled, or CBR-Luciferase/mCherry-labeled IGR-CaP1 cells in 50 μl of PBS into the left cardiac ventricle using 30G needles. Tumor growth necessitated mouse sacrifice 4.5-5.5 weeks post injection. Further details of organ harvest and processing are presented in Supplementary Methods.

### Bioluminescence imaging

*In vivo* bioluminescence imaging (BLI) was performed on the weeks indicated on an IVIS Lumina (PerkinElmer, Waltham, MA; Living Image 3.2, 1min or 1sec exposure, bin8, FOV12.5cm, f/stop1, open filter). Mice were injected intraperitoneally with D-luciferin (150mg/kg in PBS; Gold Biotechnology, St. Louis, MO) and both dorsal and ventral sides were imaged 10min later using isoflurane anesthesia (2% vaporized in O_2_). Total photon flux (photons/sec) was measured from fixed regions of interest (RIOs) over the entire mouse using Living Image 2.6.

### Tissue harvest and section preparation

Four-five weeks post tumor and 72 hour post Ad vector intravenous injection, mice were anesthetized with 2.5% 2, 2, 2-tribromoethanol (Avertin, Sigma-Aldrich, St. Louis, MO), perfused via the left ventricle with phosphate-buffered saline (PBS) followed by 10% neutral buffered formalin. Bones and organs were harvested and processed as detailed further in Supplementary Methods.

### Histochemical and immunofluorescence staining

Details regarding immunofluorescence are presented in Supplementary Methods.

### MicroCT

Methods and details of bone processing and imaging for microCT are described in Supplementary Methods.

### Immunoblotting

Overall methods of protein extract preparation were similar to previous work [39] and provided in detail in Supplementary Methods.

### Imaging/microscopy techniques and microscope/objective specification

Fluorescence and bright field microscope images were collected using a DP80 dual color/monochrome sensor CCD camera (Olympus America, Center Valley, PA) with CellSens Dimension software (Olympus Soft Imaging Solutions) with Extended Focal Imaging (EFI) function. Wide-filed images were also collected using defined scanning area mode with multiple image alignment (MIA) algorithm. Imaging experiments were repeated at least three times on independent sets of vector-injected mice. Confocal fluorescence microscope images were collected using an Olympus FV1000 confocal microscope equipped with an UPlanApo 100×/1.35 numerical aperture oil immersion objective and analyzed with Fluoview version 1.7a software (Olympus, Center Valley, PA). Collected images were processed into standard tagged image file (TIF) format using CellSens Dimension software (Olympus Soft Imaging Solutions) with Extended Focal Imaging (EFI) function.

Further Materials and Methods details are provided in the Supplementary Information.

## SUPPLEMENTARY MATERIALS FIGURES AND TABLES



## Abbreviations

PCa: Prostate cancer
AVPCa: aggressive variant prostate cancer
AR: androgen receptor
CSCs: cancer stem cells
ECs: endothelial cells
NSG: NOD.Cg-Prkdc^scid^ Il2rg^tm1Wjl^/SzJ (NSG)
Ad: adenoviral
GEM: genetically engineered mice
CAR: Coxsackie adenovirus receptor
PBS: phosphate-buffered saline
EFI: Extended Focal Imaging
MIA: multiple image alignment
CBR: click beetle red
PLK1: polo-like kinases
EMT: Epithelial-mesenchymal transition
RSPO1: R-spondin-1
CYP17A1 17α-hydroxylase/17,20 lyase: cytochrome P450 17A1
TRAP: tartrate-resistant acid phosphatase
PSMA: prostate specific membrane antigen
