# Supplementary material for: A new model of multi-visceral and bone metastatic prostate cancer with perivascular niche targeting by a novel endothelial specific adenoviral vector
Source: Oncotarget. 2017 Jan 17;8(7):12272–89. doi: 10.18632/oncotarget.14699 (PMC5355343; doi:10.18632/oncotarget.14699)
Supplement: Supplementary file 1 [file oncotarget-08-12272-s001.pdf]

A new model of multi-visceral and bone metastatic prostate cancer with perivascular niche targeting by a novel endothelial specific adenoviral vector

SUPPLEMENTARY DATA

SUPPLEMENTARY MATERIALS AND METHODS

Cell lines and generation of IGR-CaP1:CBR-LUC/mCherry reporter line

IGR-CaP1 cells were cultured in DMEM containing 10% FBS with penicillin/streptomycin/amphotericin Human prostate cancer LNCaP cells were grown in RPMI 1640 medium with 10% FBS containing penicillin/streptomycin/amphotericin B. Human prostate cancer C4-2B (a derivative line from the parental LNCaP cells), and DU145 were grown in the same DMEM growth medium for IGR-CaP1 cells. Human prostate cancer PC3 cells were grown in F-12K Medium containing 10% FBS with penicillin/streptomycin/amphotericin B. IGR-CaP1:CBR-LUC/mCherry reporter cells were created using lentiviral infection. A dual cistronic click beetle red luciferase (CBRLuc)-mCherry reporter (FUW-CBR-mCherry) was a generous gift from David Piwnica-Worms and the WU-MDACC Molecular Imaging Center). The human histone H2B and monomeric red fluorescent protein (H2B-RFP) fusion construct was obtained from Addgene [catalog # 26001], (gifted by Elaine Fuchs). The labeled cells were sorted for positive fluorochrome expression with a MoFLO high speed cell sorter by Siteman Cancer Center Flow Cytometry Core. To detect and compare prostate cancer cell responsiveness to RSPO1 and Wnt3A stimulation, one million C4-2B or IGR-CaP1 cells were seeded in six-well plates in standard culture medium, cultivated for 6 hours, and treated with 200 ng/ml of recombinant human RSPO1 or 200 ng/ml of recombinant human Wnt3A (#4645-RS-025 and #5036-MN, R&D Systems) for 16 hours before subjecting to Immunoblotting analysis.

Tissue harvest and processing

Mice where placed under deep isoflurane anesthesia. The thorax was opened, a 21 gauge cannula connected to a Perfusion One System (Leica, Buffalo Grove, IL) was inserted into the left ventricle, and the mouse was perfused with 10% formalin. Organs were removed and cut into 1 to 2 mm-thick slices. Bones were carefully dissected to remove most attached muscle and other tissues. Organ slices and bones underwent 1 hour temperature controlled microwave fixation using a Pelco Biowave processor (Ted Pella, Redding, CA) using a low variable wattage setting with sample temperature maintained at 33°C for 1 hour. Bones were decalcified for 14 days with daily changes of fresh 13% EDTA (pH7.2) solution. Harvested organ tissues were prepared in dual sets and frozen or paraffin embedded. For frozen sections, organ slices and decalcified bones were cryo-preserved in 30% sucrose in PBS at 4°C overnight, embedded in NEG50 mounting medium (Thermo Fisher Scientific, Waltham, MA), and frozen in a liquid nitrogen pre-chilled 2-methylbutane-containing glass beaker. Fixed soft tissues and decalcified bones were also switched to graded alcohols and xylenes and embedded in paraffin.

MicroCT

Dissected spines and hindlimbs were scanned using X-ray microtomography (Scanco uCT40, Brüttisellen, Switzerland) at 70kVp/144uA at a nominal resolution of 10um with an integration time of 300ms. DICOM images were exported for use in OsiriX VERSION# (Pixemo SARL, Bernex, Switzerland) for visualization. Three dimensional volume renderings were generated using no color look-up table, default shading settings, and window location and width of 2422 and 1372, respectively. The 3D images were then smoothed with a 3x3 blur filter and the crop tool utilized to isolate regions and views of interest.

Histochemical and immunofluorescence staining

For histochemical staining, 5-µm paraffin block sections were prepared. All harvested organs were subject to histological analysis of metastasis distribution by hematoxylin and eosin staining. Tartrate-resistant acid phosphatase (TRAP) histochemical assay, an osteoclast marker, and Masson’s trichrome staining for osteoid detection indicative of new bone formation were performed by the Histology and Morphometry Core of the Musculoskeletal Research Center at the Washington University School of Medicine. For immunofluorescence, frozen lung and bones were cryo-sectioned at 10 µm, and slices from other organs were cut at 16 m. Sectioning of frozen bones was carried out

using the CryoJane taping system (Leica Biosystems Inc., Buffalo Grove, IL). Frozen section slides were air-dried for ten minutes and washed three times in PBS to remove NEG50 mounting medium. Paraffin section slides were deparaffinized in xylene and rehydrated in alcohol and then water. Both frozen and paraffin section slides were then incubated with protein block solution (5% donkey serum and 0.1% Triton X-100 in PBS) at room temperature for one hour and then at 4°C in protein block containing primary antibodies overnight. Primary antibodies used in this study included rat anti-endomucin 1:1,000 (#14-5851-81, eBioscience, San Diego, CA), Armenian hamster anti-CD31 1:1,000 and rabbit anti-CXCR4 1:200 (#MAB1398Z and #AB1846, EMD-Millipore, Billerica, MA), and chicken anti-GFP 1:400 (#A-11122, Thermo Fisher Scientific), rabbit anti-E cadherin 1:200 (ab40772, Abcam, Cambridge, MA), goat anti-vimentin 1:20 (sc7557, Santa Cruz Biotechnology Inc., Dallas, TX), rat anti-CD44 1:50 (#550538, BD Biosciences, San Jose, CA), mouse anti-SDF1 1:20 (#MAB350, R&D Systems Inc., Minneapolis, MN), rabbit anti-osteocalcin (ab93876, Abcam). On day 2, the slides were washed three times in PBS, incubated with corresponding 1:400 diluted aminomethylcoumarin (AMCA-, Alexa Fluor 488-, or Alexa Fluor 594-conjugated secondary antibodies (Jackson ImmunoResearch Laboratories, West Grove, PA), and counterstained with SlowFade Gold Antifade mounting reagent with or without 4,6-diamidino-2-phenylindole (DAPI) (Thermo Fisher Scientific).

Immunocytochemistry

Log-phase IGR-CaP1 cells grown in Nunc Lab-Tek 8-well Chamber Slides (Sigma Aldrich) were treated directly within the wells throughout the staining procedure. In brief, PBS-washed cells were fixed in 50% acetone/50% methanol for 30 minutes on ice, washed three times with PBS, blocked with protein block solution described above for one hour at room temperature, and then at 4°C in protein block containing primary antibodies overnight. Primary antibodies used in this study included rabbit anti-synaptophysin 1:200 (GTX100865, GeneTex), rabbit anti-Ki67 1:250 (ab15580, Abcam), rabbit anti-P21 1:250 (sc397, Santa Cruz Biotechnology), rabbit anti-P53 1:400 (#2527, Cell Signaling Technology), mouse anti-NCAM 1:200 (#3576, Cell Signaling Technology), and rabbit anti- gammaH2A.X 1:100 (#9718, Cell Signaling Technology). Following 3 washes with PBS, the secondary antibody treatment was identical to immunofluorescence staining described above. For nucleus counterstaining, cells were incubated in PBS containing 1 μM TO-PRO-3 (Thermo Fisher Scientific) for 30 minutes at room temperature, followed by three washes with PBS. Removal of chamber gasket was performed according to manufacturer's instruction, and the slides were mounted with with SlowFade Gold Antifade mounting reagent without 4,6-diamidino-2-phenylindole (DAPI).

Immunoblotting

T-75 flasks containing late log-phase human prostate cancer cells were washed once with cold PBS, and lysed on ice with 500 μl cold radio-immuno-precipitation assay buffer (RIPA; 20 mM Tris-HCl [pH 7.6], 0.15 M NaCl, 1% sodium deoxycholate, 1% NP40, 1 mM EDTA, 1 mM EGTA) supplemented with Protease Inhibitor Cocktail, 1:50 (Sigma-Aldrich, St. Louis, MO). Protein lysates were quantified for protein concentration using the Pierce BCA Protein Assay Kit (Thermo Fisher Scientific). Protein samples with same amount protein (30 g) were separated on polyacrylamide gels and transferred to polyvinylidene difluoride (PVDF) membranes. Protein loading in individual lanes was further normalized to β-tubulin content. Membranes were blocked in 5% nonfat dry milk in Tris-buffered saline containing 0.5% Tween 20 (TBST, pH 7.6) and incubated in 5% BSA in TBST containing the primary antibodies overnight. The primary antibodies included rabbit anti-Notch1 1;1000 (#3608, Cell Signaling Technology, Danvers, MA), rabbit anti-Notch2 1:1000 (#5732, Cell Signaling Technology), rabbit anti-Notch3 1:1000 (#5276, Cell Signaling Technology), rabbit anti-Jagged1 (#2620, Cell Signaling Technology), rabbit anit-Jagged2 1:1000 (#2210, Cell Signaling Technology), rabbit anti-Dll4 1:1000 (#2589, Cell Signaling Technology), rabbit anti-TACE/ADAM17 1:1000 (#6978, Cell Signaling Technology), rabbit anti-cleaved Notch1 intracellular domain (Val1744) 1:1000 (#4147, Cell Signaling Technology), rabbit anti-RBPJ 1:1000 (#5313, Cell Signaling Technology), rabbit anti-Axin2 1:1000 (#ab109307, Abcam), rabbit anti-Wnt3a 1:1000 (#2721, Cell Signaling Technology), rabbit anti-Wnt5a/b 1:1000 (#2530, Cell Signaling Technology), goat anti-Wnt2 (#AF3464, R&D Systems), rabbit anti-Cyclin D1 1:1000 (#2978, Cell Signaling Technology), rabbit anti-androgen receptor 1:200 (sc-816, Santa Cruz Biotechnology Inc.), rabbit anti-PSMA 1:1000 (#12815, Cell Signaling Technology), rabbit anti-Synaptophysin 1:200 (GTX100865, GeneTex), mouse anti-NCAM/CD56 1:1000 (#3576, Cell Signaling Technology), rabbit anti-N-Cadherin 1:1000 (#13116, Cell Signaling Technology), rabbit anti-c-Myc 1:1000 (#5605, Cell Signaling Technology), rabbit anti-pRB 1:1000 (#9313, Cell Signaling Technology), rabbit anti-Aurora A 1:1000 (#4718, Cell Signaling Technology), rabbit anti-PLK1 1:1000 (#4513, Cell Signaling Technology), rabbit anti-P53 1:1000 (#2527, Cell Signaling Technology), rabbit anti-P21 1:200 (sc-397, Santa Cruz Biotechnology

Inc.), rabbit anti-p-P21^{T145} 1:200 (sc-20220, Santa Cruz Biotechnology Inc.), rabbit anti-Bcl-xL 1:1000 (#2764, Cell Signaling Technology), rabbit anti-Bcl2 1:1000 (#2876, Cell Signaling Technology), rabbit anti-P-histone H2A.X^{S139} 1:1000 (#9718, Cell Signaling Technology), rabbit anti-HGF 1:200 (ab83760, abcam), rabbit anti-P-c-Met$^{Y1234/1235}$ 1:1000 (#3129, Cell Signaling Technology), rabbit anti-cMet 1:1000 (#4560, Cell Signaling Technology), rabbit anti-P-Jak1$^{Y1022/1023}$ 1:1000 (#3331, Cell Signaling Technology), rabbit anti-Jak1 1:1000 (#3332, Cell Signaling Technology), rabbit anti-P-Jak2$^{Y1007/1008}$ 1:1000 (#3771, Cell Signaling Technology), rabbit anti-JAK2 1:1000 (#3229, Cell Signaling Technology), rabbit anti-P-STAT3^{Y705} 1:1000 (#9145, Cell Signaling Technology), rabbit anti-P-STAT3S727 1:1000 (#9134, Cell Signaling Technology), rabbit anti-STAT3 1:1000 (#9132, Cell Signaling Technology), rabbit anti-P-c-RAF^{S338} 1:1000 (#9427, Cell Signaling Technology), rabbit anti-C-RAF 1:500 (sc-227, Santa Cruz Biotechnology, Inc.), rabbit anti-P-MEK1/2^{S221} 1:1000 (#2338, Cell Signaling Technology), rabbit anti-MEK1/2 1:1000 (#9126, Cell Signaling Technology), rabbit anti-P-ERK1/2$^{T202/Y204}$ 1:1000 (#4376, Cell Signaling Technology), rabbit anti-ERK1/2 1:1000 (#9102, Cell Signaling Technology), rabbit anti-ZEB1 1:5000 (#3396, Cell Signaling Technology), rabbit anti-Twist1/2 1:2000 (GTX127310, GeneTex Inc.), rabbit anti-Snail 1:1000 (#3879, Cell Signaling Technology), rabbit anti-Slug 1:1000 (9585, Cell Signaling Technology), rabbit anti-E-Cadherin 1:1000 (#3195, Cell Signaling Technology), rabbit anti-Vimentin 1:1000 (#5741, Cell Signaling Technology), and rabbit anti-β-tubulin 1:20,000 (Novus Biologicals, Littleton, CO). Membranes were washed three times with TBST and incubated in TBST containing 5% milk with the corresponding IgG-horseradish peroxidase conjugate, 1:5,000, (all from Santa Cruz Biotechnology, Santa Cruz, CA) for 1 hour. After three TBST washes, peroxidase activity was revealed by enhanced chemiluminescence using ECL2 or SuperSignal West Femto Western Blotting Substrate (both from Thermo Fisher Scientific) and imaged using a Chemidoc XRS imaging system (Bio-Rad Laboratories, Hercules, CA).

Vector construction and administration

The plasmid encoded expression cassette comprised of the *ROBO4* enhancer/promoter element coupled to the enhanced green fluorescent protein *EGFP* gene, followed by the bovine growth hormone polyadenylation signal. The expression cassette was cloned into a shuttle plasmid (pShuttle, Qbiogene, Carlsbad, CA) and confirmed using restriction enzyme mapping and partial sequence analysis. The shuttle plasmid was linearized with *Pme* I enzyme and integrated into the viral genome by homologous recombination with pVK503C plasmid, containing a gene encoding CDCGRDCFC peptide inserted into the HI-loop of the Ad fiber knob protein in *E. coli* strain BJ5183. To generate hexon-modified RGD.H5/3.ROBO4-EGFP vector, the *SexA* I - *Hpa* I digest product of adenovirus serotype 5 hexon was substituted with a 618 bp fragment from adenovirus serotype 3 hexon (nucleotides 1127-1745). The insert sequences were confirmed by partial sequencing analysis. Recombinant viral genomes were packaged into virus particles following transfection of HEK293 cells using SuperFect transfection reagent (QIAGEN, Chatsworth, CA). RGD.H5/3.ROBO4-EGFP virus was propagated in HEK293 cells, purified twice by CsCl gradient centrifugation and dialyzed against phosphate-buffered saline, pH 7.4 with 10% glycerol. The viral particle (vp) concentration was determined by absorbance of dissociated virus at A_{260} nm using a conversion factor of 1.1×10^{12} vp per absorbance unit. Two to three weeks post tumor cell injection; mice were administrated with $1x10^{11}$ particles of adenovirus in 200 L of saline via tail-vein injection. Seventy-two hours post virus injection, mice were anesthetized and tissues harvested for histochemical and immunofluorescence processing.

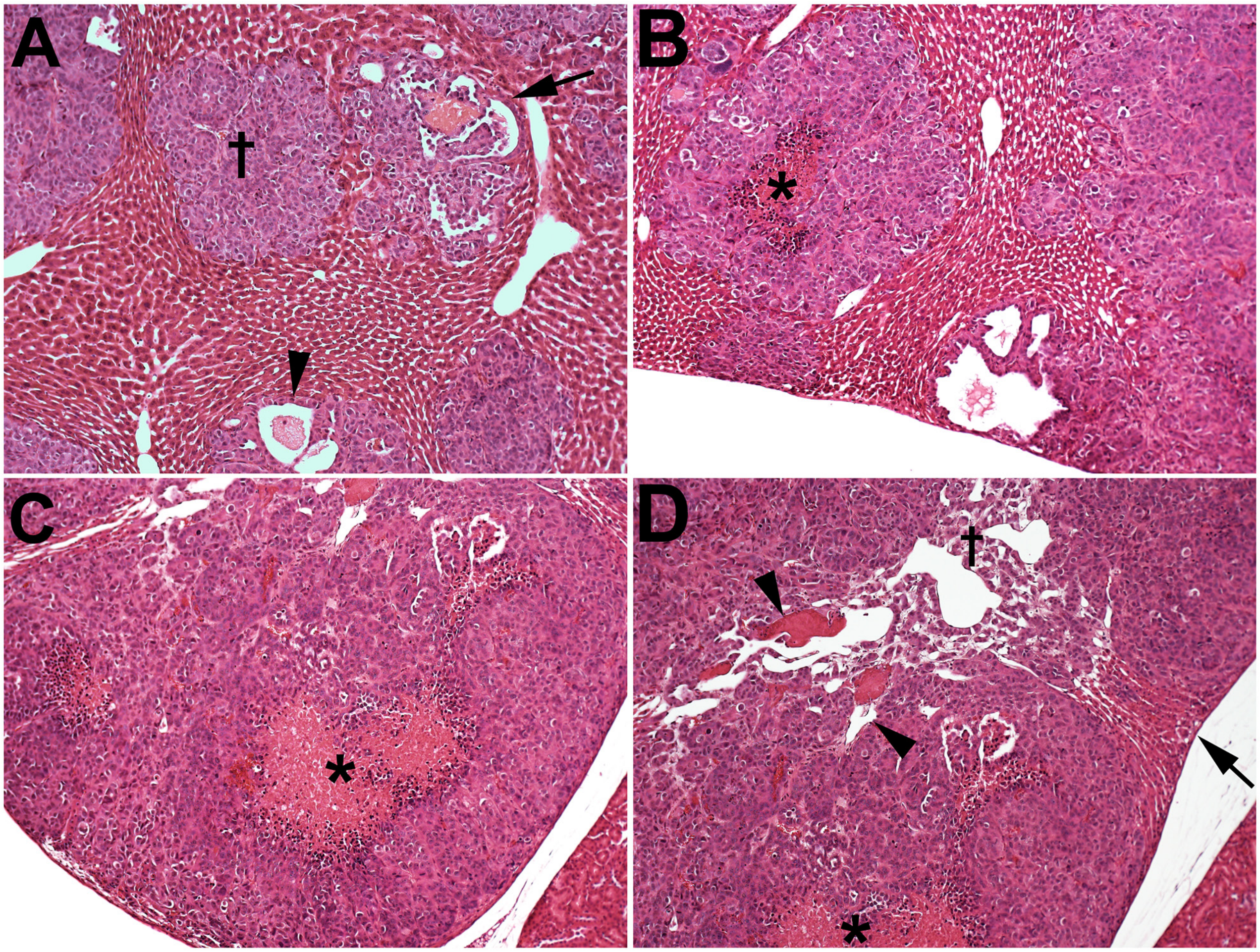

Supplementary Figure 1: Multiplex IGR-CaP1 histopathology. A. Liver tumors containing relatively circumscribed sheets of poorly differentiated cells (Latin cross), pseudo-papillary (arrow) and glandular architecture (arrowhead). **B.** Liver tumor with central/comedo necrosis (asterisk). **C.** Adrenal tumor (also D) almost completely replaces host glandular tissue. Region displayed also contains central/comedo necrosis (asterisk). **D.** Region adjacent to C reveals intravenous tumor thrombi (arrowheads). There is residual adrenal medulla (Latin cross) and cortex (arrow).

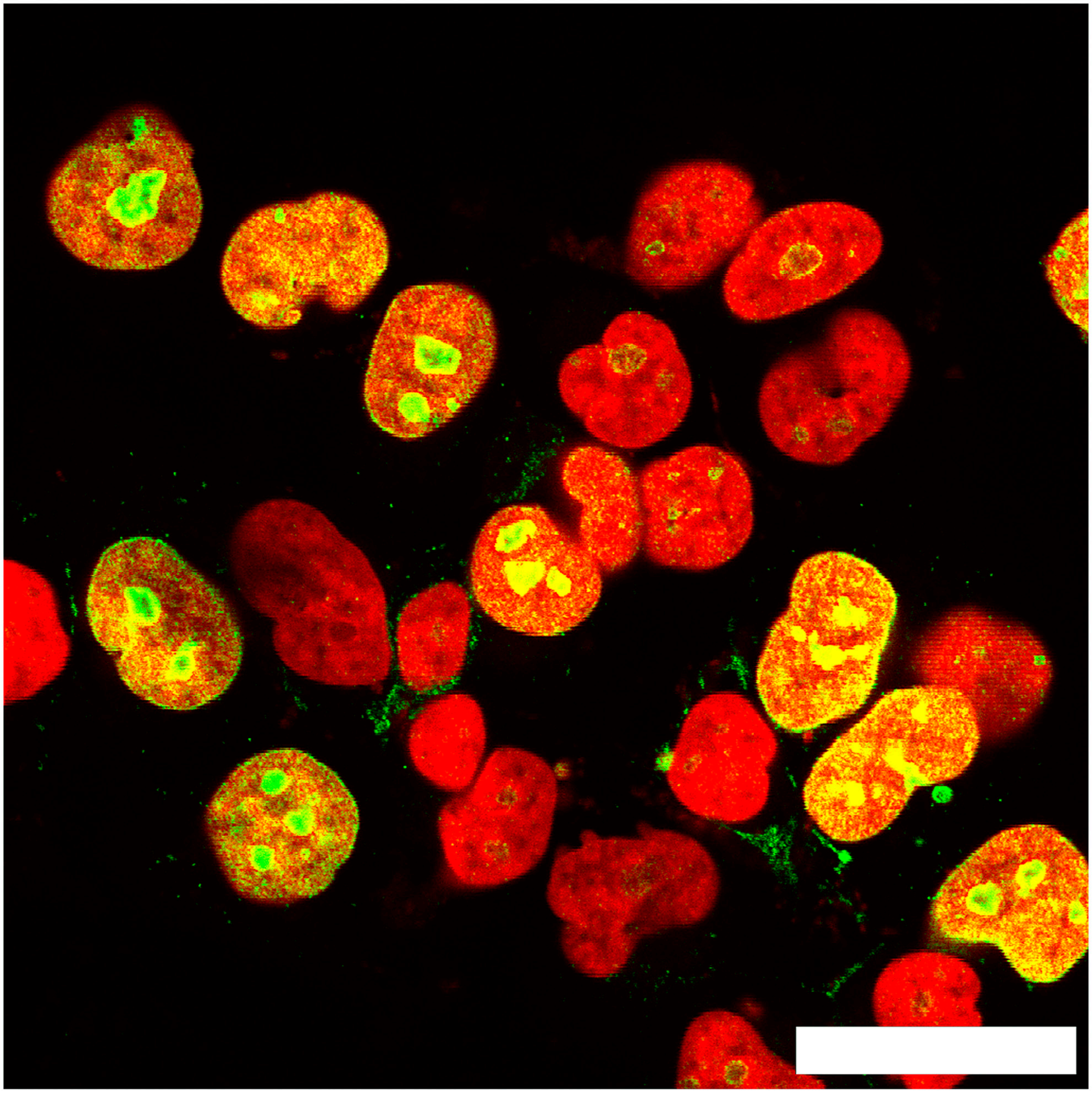

Supplementary Figure 2: Marked upregulation of proliferation activity in IGR-CaP1 cells. High level diffuse nuclear and focal perinucleolar Ki67 expression (Green) in cultured IGR-CaP1 cells. Red: TOPRO3 nuclear counterstain. Bar: 20 µm.

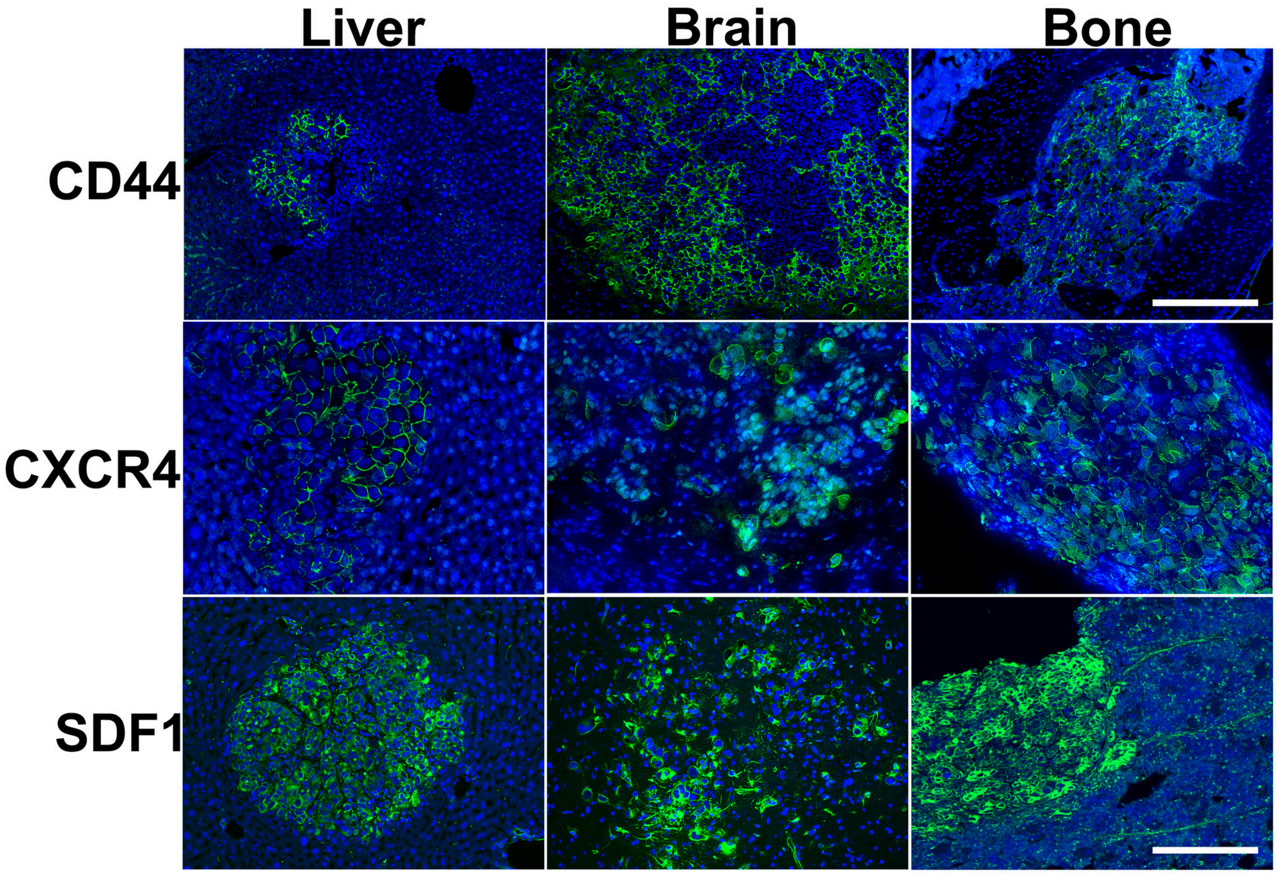

Supplementary Figure 3: Enhanced expression of cancer stem cell niche adhesive mediators in metastatic target organs. CD44 and CXCR4 are upregulated and plasma membrane localized in liver, brain and bone IGR-CaP1 experimental metastases. Marked induction of cytoplasmic SDF1 is evident in metastases in the same organs with additional expression detected in bone marrow reticular cells, and arterioles Bar: Top row: 400 µm; Middle and bottom rows: 200 µm.

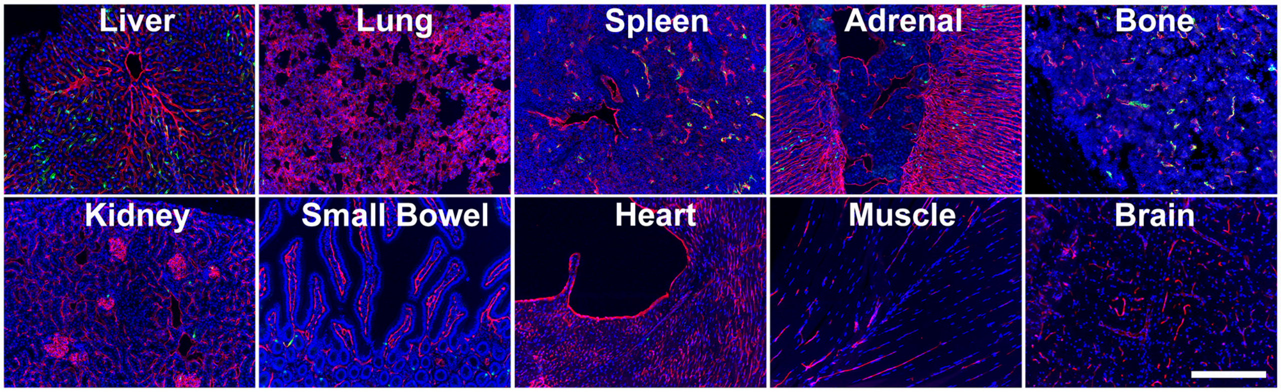

Supplementary Figure 4: Low-level RDG.H5/3.ROBO4 vector expression in a limited subset of visceral organs and bone marrow in nontumor bearing mice. Scattered expression of the vector in the liver sinusoidal, splenic, and bone marrow vascular endothelial cells four days post intravenous virus injection. Adrenal cortical cell expression is also evident. Near absent expression in lung, small bowel, and kidney vasculature, with no detectable expression in heart, muscle, and brain vascular endothelial cells. Red: endomucin/CD31 cocktail; Green: EGFP; Blue: DAPI. Bar: 1 mm all panels.
